# Haematopoietic development and HSC formation *in vitro*: promise and limitations of gastruloid models

**DOI:** 10.1042/ETLS20230091

**Published:** 2023-12-14

**Authors:** Liza Dijkhuis, Ayona Johns, Denise Ragusa, Susanne C. van den Brink, Cristina Pina

**Affiliations:** 1 Department of Hematopoiesis, Sanquin Research, 1066 CX Amsterdam, The Netherlands; 2 College of Health, Medicine and Life Sciences, Brunel University London, Uxbridge UB8 3PH, U.K.; 3 Centre for Genome Engineering and Maintenance, Brunel University London, Uxbridge UB8 3PH, U.K.; 4 Program in Cancer Research, Hospital del Mar Medical Research Institute, 08003 Barcelona, Spain

**Keywords:** gastruloid models, haematopoietic stem cells, pluripotent stem cells

## Abstract

Haematopoietic stem cells (HSCs) are the most extensively studied adult stem cells. Yet, six decades after their first description, reproducible and translatable generation of HSC *in vitro* remains an unmet challenge. HSC production *in vitro* is confounded by the multi-stage nature of blood production during development. Specification of HSC is a late event in embryonic blood production and depends on physical and chemical cues which remain incompletely characterised. The precise molecular composition of the HSC themselves is incompletely understood, limiting approaches to track their origin *in situ* in the appropriate cellular, chemical and mechanical context. Embryonic material at the point of HSC emergence is limiting, highlighting the need for an *in vitro* model of embryonic haematopoietic development in which current knowledge gaps can be addressed and exploited to enable HSC production. Gastruloids are pluripotent stem cell-derived 3-dimensional (3D) cellular aggregates which recapitulate developmental events in gastrulation and early organogenesis with spatial and temporal precision. Gastruloids self-organise multi-tissue structures upon minimal and controlled external cues, and are amenable to live imaging, screening, scaling and physicochemical manipulation to understand and translate tissue formation. In this review, we consider the haematopoietic potential of gastruloids and review early strategies to enhance blood progenitor and HSC production. We highlight possible strategies to achieve HSC production from gastruloids, and discuss the potential of gastruloid systems in illuminating current knowledge gaps in HSC specification.

## Introduction

Mammalian haematopoietic stem cells (HSCs) arise from a specialised haemogenic endothelium (HE) in the ventral wall of the dorsal aorta, approximately mid-gestation (E10.5–E11.5) in mouse and 32 days post-fertilisation (Carnegie Stage, CS14–15) in humans [1,2]. HSC emergence is a late event in the haematopoietic specification in the embryo. The first haematopoietic cells are generated in the blood islands of the yolk sac (E7.5 in mouse; CS6–7 in human) [3–5]: they are primitive erythrocytes and macrophages, and serve oxygenation, tissue remodelling and early defence needs of the growing embryo. Two additional waves of haematopoiesis ensue. These are characterised by haematopoietic cell formation from a specialised HE in a process of endothelial-to-haematopoietic transition (EHT) [6,7]. The first subsequent wave of blood production, still localised to the yolk sac at E8.5–9.5 in mouse and CS13–15 in human, generates erythro-myeloid progenitors (EMP) that differentiate into erythrocytes, megakaryocytes (platelet-precursors), macrophages and neutrophils [2,8,9]. The final wave of haematopoiesis occurs in the embryo proper, specifically in the ventral wall of the dorsal aorta and adjacent large vitelline arteries in the aorta–gonad–mesonephros (AGM) region (mouse E9.5–E11.5; CS13–14 in human) [2,10–13]. This final wave generates myelo-lymphoid progenitors (MLPs) with the potential to differentiate into neutrophils, macrophages and lymphocytes [14]. Lastly, it generates multipotent progenitors (MPPs) and HSC, which is limited to a time-window between E10.5 and E11.5 in mouse (CS14–15 in human), after which new haematopoietic specification ceases [12].

Blood cells produced at extra-embryonic and intra-embryonic locations migrate to the developing foetal liver (FL), where they proliferate and differentiate to sustain the blood needs of the embryo [15,16]. Importantly, the mature blood cell types used by the embryo during development are the progeny of the uni- and multilineage progenitors formed in the yolk sac and AGM, with minimal or no participation from HSC [17]. Instead, HSC expand in the FL and eventually migrate to the developing bones to establish themselves in the bone marrow (BM) niche of the bone cavities. The extent to which HSC expand in the FL vs. the BM, and differences between foetal and adult HSC are not fully resolved [18]. Foetal progenitors cease their contribution to blood production in the perinatal period [19]. HSC-driven haematopoiesis starts in the first month post-natally in the mouse, and in late gestation in the human, marking the start of long-term, life-long adult haematopoiesis [20,21].

Unlike highly-proliferative foetal HSCs, adult HSCs are mostly considered to be quiescent [20,22,23], as the majority of the long-term engraftable HSCs reside in the G0 cell cycle phase [24,25]. Adult HSCs can become activated upon stimulation by environmental factors and start differentiating towards MPPs [26]. Individual HSCs can take various differentiation routes as they progressively restrict their differentiation potential, with the eventual generation of the different mature blood cell types [27,28]. HSC themselves are heterogeneous and present distinct myelo-lymphoid lineage biases, with the process of diversification initiated during development [29].

Haematopoietic and endothelial cells are derivatives of the lateral plate mesoderm (LPM), which also generates the heart and blood vessels. Specification of HSC in the dorsal aorta and adjacent arteries positions HSC precursors at the posterior of the LPM. The anterior LPM is involved in the specification of the heart, but it also produces endothelial and blood cells, namely the first progenitors in the yolk sac [30]. Specification of HE and haematopoietic cells requires signals from adjacent structures, namely the neural tube (WNT), the notochord (Shh) and paracrine signals from adjacent LPM and intermediate mesoderm (IM) derivatives (BMP4, Noggin, FGF2, VEGF, SCF) [31]. HSC specification is also dependent on the developing autonomous nervous system [32], which are neural crest derivatives [33].

The above summary of cellular and chemical components required for HSC specification is by no means exhaustive and has been reviewed extensively elsewhere [11,34]. Nevertheless, it highlights the complexity of the haematopoietic and HSC microenvironments during development, suggesting that effective recapitulation of HSC production *in vitro* requires or benefits from effective reconstitution of the accompanying niche. In principle, time-adjusted reconstitution of tissue architecture may also support the production of successive waves of blood production for the correct specification of HSC. Here, we review the emergent gastruloid models of haematopoiesis and HSC production and explore future directions of protocol optimisation.

## Current *in vitro* models of embryonic haematopoiesis and HSC production

Adult HSCs sustain life-long blood production by balancing rare self-renewal and differentiation divisions and are capable of replenishing every blood cell lineage [35]. This feature underlies their use in HSC transplants to reconstitute the haematopoietic system in treatments for a wide range of congenital and acquired haematopoietic disorders, as well as BM re-cellularisation post-chemotherapy for haematological and non-haematological malignancies [36]. However, the limited availability of HSC donors and persisting limitations in expanding and maintaining HSCs *in vitro* decrease therapy availability and efficacy [37].

Generation of *de novo* HSC from embryonic sources or patient-derived pluripotent cells would greatly improve the availability of HSC transplants. Embryonic stem cells (ESCs) [38,39] and embryonic-like induced pluripotent stem cells (iPSC) (generically, PSCs) [40,41] can be maintained long-term in culture with preserved potential and would thus constitute a virtually unlimited source of HSCs as well as their differentiated progeny. Importantly, PSCs obtained directly from the respective patient or engineered to remove histocompatibility antigens provide autologous or autologous-like sources of HSCs for the production of universal transplantation and transfusion products [42]. Furthermore, PSC can potentially constitute a source of developmentally younger transplantable HSCs which minimise the frequency of epigenetic and genetic changes associated with HSC ageing that can modify or compromise HSC functionality [43]. Correct HSC production from PSC sources requires systematic understanding of processes and requirements of HSC specification and functional maturation *in vivo,* in order to establish *in vitro* models that faithfully mimic this process.

**Figure 1.**
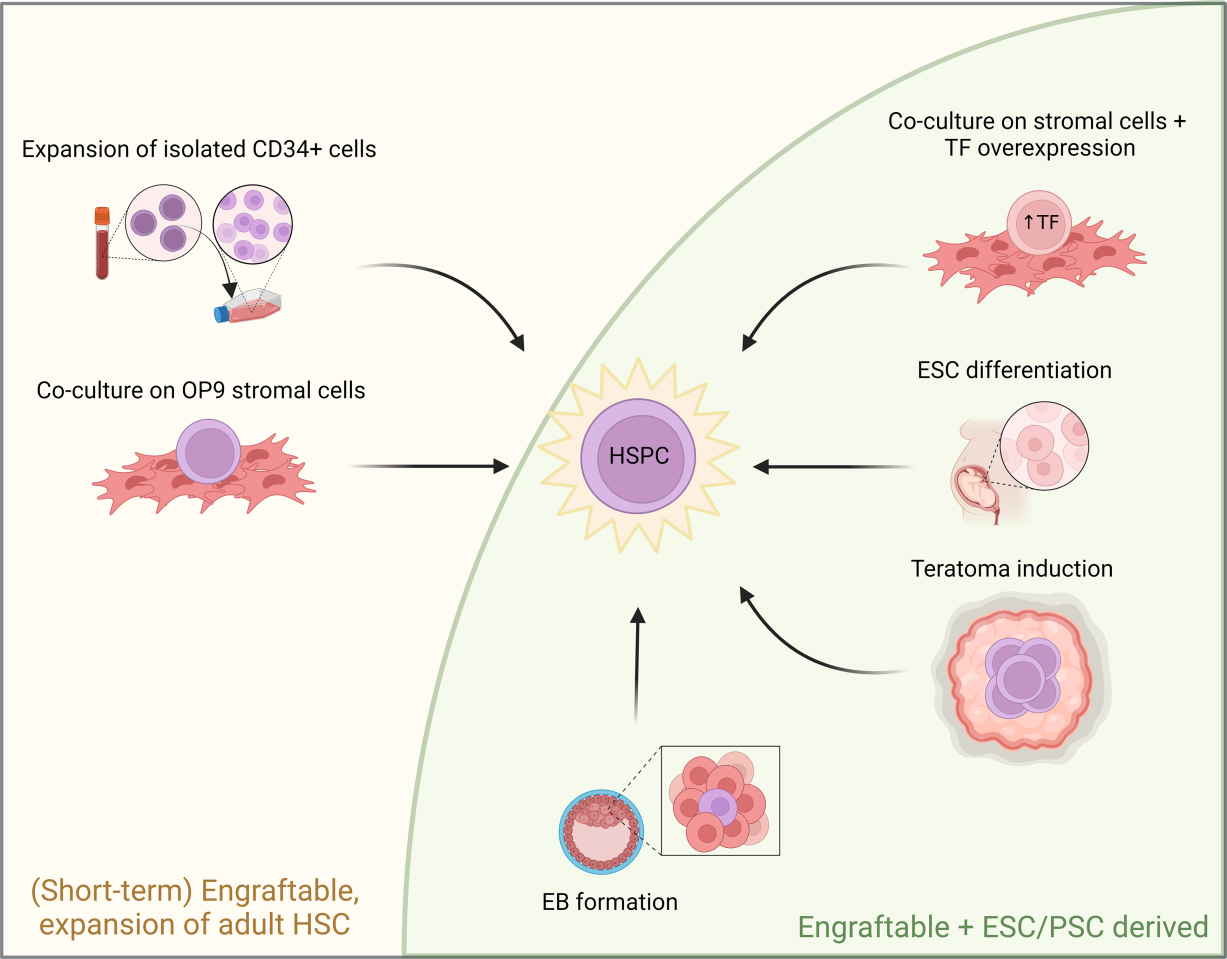
Schematic diagram of approaches to generate HSCs. Methods able to generate *de novo* transplantable hematopoietic stem and progenitor cells (HSPC) from ESCs/PSCs remain rare. Most approaches rely on mimicking HSC-niche interactions (via teratoma or embryoid body (EB) induction) and/or overexpression of transcription factors (TFs). Protocols that do not achieve engraftment (e.g. OP9-co-culture approaches by themselves do not support long-term transplantation potential) and those that do not use embryonic/pluripotent material as a starting point (e.g. the use of CD34+ cells isolated from peripheral blood) (envisioned in the left part of the figure) are out of the scope of our review and are noted here for completion only. Image is created with Biorender.com.

Over the last few decades, many attempts have been made to generate culture systems that recapitulate embryonic haematopoiesis [44] (Figure 1, with approaches that generated engraftable hematopoietic stem and progenitor cells (HSPCs) in Table 1). Production of HSCs is judged by the ability of the cells generated in culture to reconstitute the entire haematopoietic system of an irradiated and/or immune-deficient mouse long-term (experimentally, >4–6 months), whilst maintaining functional HSCs which can successfully reconstitute a secondary mouse host upon serial transplantation [45]. Table 2 lists large-scale protocols of translational potential that make use of PSCs to generate differentiated blood cells for transfusion purposes.

**Table 1.** Current approaches generating transplantable HS(P)Cs

Output	Source	Approach	Publication
HSPCs	Human iPSCs	*In vivo* teratoma induction	[63,64]
Definitive HSPCs	Primitive progenitors from the yolk sac or ESCs	Ectopic expression of HoxB4 and co-culture with OP9 stromal cells	[65]
HSPCs	Mouse ESCs/iPSCs	Ectopic expression of Lhx2 in ESC/iPSC-derived mesoderm	[67]
HSPCs	Human PSCs	Morphogen-driven differentiation and ectopic TFs for cell fate conversion	[66]

**Table 2.** Large-scale blood cell production from ESC or PSC source

Output	Source	Approach	Publication
Erythroid, megakaryocytic and myeloid cells	Human induced pluripotent stem cells (hiPSCs)	Feeder-free, monolayer differentiation system starting from a single hematopoietic initiation step	[58]
Platelets	Human cord blood (CB) CD34+cells	Co-culturing on hTERT stromal cells in a three-phase system	[59]
Universal platelets	hiPSCs	Knocking-out β2 microglobulin in a sequential-phase system	[60]
Erythrocytes	Human CB HSCs	Liquid culture system, followed by co-culturing on human fetal liver stromal cells	[62]
Erythrocytes	Human ESCs	EB formation and hemangioblast precursor induction, followed by erythroid differentiation	[61]
Neutrophils	Human CB CD34+ cells	Four-stage culture approach in a roller bottle production platform	[70]

Most current models of PSC-based haematopoietic cell specification are based on an initial step of non-directed PSC differentiation into the three germ layers using embryoid bodies, which is followed by specific cues to promote the specification of mesoderm and the formation of vasculature [46–48]. The correct specification of HE is accepted as a key step in haematopoietic specification *in vitro* and is central to protocols which have more closely approximated HSC production [49,50]. As an alternative step to embryoid body-based differentiation, or after embryoid body and mesodermal specification steps, the models attempt to recreate the interaction with an adult-like haematopoietic niche, commonly through co-culturing of HSPCs with OP9 stromal cells [51–53]. OP9 cells were established by the transformation of bone osteoblasts from macrophage colony-stimulating factor (M-CSF)-deficient mice and enable HSPCs expansion by recapitulating interactions established between HSCs and stromal niche cells in the BM [54,55]. Other methods to recreate environmental cues at the BM site have included the use of artificial 3D scaffolds, such as polydimethylsiloxane (PDMS) matrixes. PDMS scaffolds have been shown to increase the expansion of undifferentiated HSC and maintain their pluripotency in addition to optimised growth factor conditions [56,57].

Many approaches have been successful at generating differentiated blood cells, such as erythrocytes and platelets [58–62], but the cells retain embryonic-like characteristics (e.g. the production of embryonic globin molecules, or a reduced platelet output from individual megakaryocytes), suggesting that the maturation of haematopoietic progenitors remains incomplete. Critically, the production of bona fide HSCs remains inefficient. Methodologies capable of generating HSPCs through ectopic expression of HSC-associated genes are not directly transferrable to the clinic [51,63–67]. While the production of engraftable mouse HSPC from PSC sources without overexpression of transgenes has been reported [68], there was limited or no long-term engraftment in the BM, with donor cells predominantly in the spleen and biased towards the lymphocytic lineage [69]; no secondary transplants were reported. Importantly, reports of successful generation of transplantable HSPCs correspond to the production of only a few functional HSCs. Candidate HSCs have not been characterised in terms of possible heterogeneity or lineage bias, and the impact of the identity of the programmed or reprogrammed cell remains unexplored.

Even though current approaches can in principle be optimised and up-scaled to improve HSC production, they also pose practical challenges to clinical translation due to potential off-target and oncogenic effects secondary to ectopic gene expression. Therefore, more physiological models that do not require genetic modification or co-culture with stromal cell lines which may be carried in the transplantation product are needed.

## Gastruloid models of blood formation

**Figure 2.**
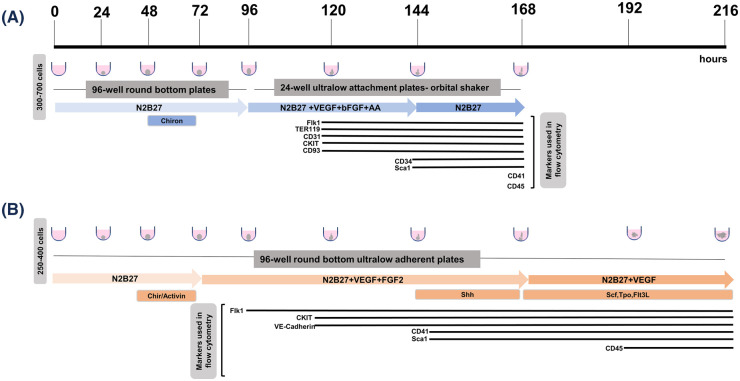
Schematic representation of current protocols that mimic developmental hematopoiesis. Black timeline represents the starting point of each protocol, up until the termination. (**A**) Protocol used by Rossi *et al.* [76], where 300–700 cells were aggregated into 96 well-round bottom plates cultured in N2B27. A pulse of CHI99021 was added between 48 and 72 h. Post-96 h, the aggregates were transferred into 24-well plates shaking on orbital shaker (as shown in grey box) and cultured in N2B27, VEGF, bFGF and AA until 14 4h. After 144 h the aggregates are cultured in N2B27 alone. Flow cytometry parameters are illustrated in black with the emergence of each marker corresponding to the timeline. (**B**) Protocol by Ragusa *et al.* [77], where 250 cells were aggregated in N2B27 into 96 well-round bottom ultra-low adherent plates as shown in grey box. A pulse of CHI99021 + Activin A is added between 48 and 72 h. Gastruloids are then cultured in N2B27, VEGF and FGF2. Shh was added between 144 and 168 h. Other growth factor cytokines (SCF, thrombopoietin — TPO, and Flt3L) are added in the last 2 days along with just N2B27 and VEGF. Flow cytometry parameters are illustrated in black with the emergence of each marker corresponding to the timeline.

Gastruloid cultures present an opportunity to generate HSCs in a single and scalable culture system without the need for genetic manipulation. Gastruloids are self-organising 3D aggregates of PSC [71,72], which have been shown to capture early events in gastrulation and tissue patterning with temporal and spatial accuracy [73,74]. Variations of gastruloid culture conditions bias the system towards different outputs [75–82] and are discussed elsewhere in this issue. For the purpose of this review, we will consider recent reports describing the blood generation potential of gastruloids [76,77], summarised in Figure 2. We will also review information on the specification of mesodermal lineages, as these are critical first events on the generation of endothelium and blood.

HSCs are derivatives of the posterior LPM, and require signals from the notochord and neighbouring LPM and IM derivatives [83,84]. LPM also generates endothelium and cardiac precursors, the latter differentiating from the anterior LPM, which also generates blood cells in the early waves of haematopoietic production [30,31]. Mouse gastruloids generated in a 5-day protocol with a 24 h-pulse of GSK3β inhibitor CHI99021 used as a WNT-agonist, have robust activation of primitive streak markers and of paraxial — pre-somitic and somitic — mesoderm [75], but limited representation of LPM and IM markers at the exception of the early blood endothelial marker FLK1/VEGFR2/*Kdr*. Although *Kdr* is also expressed by paraxial mesoderm-derived lymphatic endothelium [85], inspection of single-cell profiles of mouse gastruloids clearly separate *Kdr* from paraxial mesoderm markers such as *Pax3*, suggesting incipient LPM specification [80]. Embedding of gastruloids in a poorly-defined extracellular matrix — commercial Matrigel — structurally organises the somitic structures [75,80] and enhances expression of IM genes [75], but does not alter LPM expression. Limited representation of LPM markers is also noted in elegant embryoid experiments which juxtapose trophoectoderm and epiblast organoids or non-epithelialised gastruloids for the promotion of anterior neural structures [86]. Not dissimilar to the somitogenesis protocols, epithelialised epiblast organoids form in the presence of Matrigel, thus capturing contributions of an artificial extracellular matrix to embryo patterning. While posterior LPM programmes are minimally affected by the epiblast-like epithelialisation in Matrigel, anterior cardiogenic mesoderm is inhibited by the latter, as is definitive endoderm [86]. In contrast, the pronephros derivative of IM is more extensively represented in embryoids started from epiblast organoids, compatible with the up-regulation of IM programmes upon Matrigel embedding. Important for HSC specification, neural crest is also promoted, although not strictly dependent on, Matrigel-containing epiblast-forming conditions [32]. Altogether, these results demonstrate that it is possible to promote the formation of tissues that are relevant to haematopoiesis, such as LPM, IM and neural crest, in gastruloids by modifying their culture protocol.

The presence of incipient endothelial programmes, as highlighted by *KDR/VEGFR2* expression, extends to human gastruloids [87], and is indicative of the potential to initiate haematopoietic programmes. In mouse, the addition of TGFβ-inhibitor Activin A to CHI99021 during the gastruloid patterning pulse, results in strong and polarised activation of a *Flk1* (*Kdr*)*-GFP* reporter, which gets further patterned into a still disorganised endothelial network upon exposure to FGF2 and VEGF [77]. Activin A is not required for endothelial or cardiac specification, and can promote endodermal fates under specific culture conditions [76,78]. Nevertheless, we observed increased frequency of *Kdr*-expressing cells upon Activin A pulse, coincident with a specific requirement for *Flk1-GFP* reporter expression, and indeed blood formation, under culture conditions used in Ragusa *et al.* [77].

Addition of FGF2 and VEGF after the patterning pulse supported endothelial, cardiogenic and haemogenic programmes in mouse gastruloids [76–78]. Rossi *et al.* [78] exposed the developing gastruloid to FGF2 and VEGF in the presence of ascorbic acid and mechanical flow through orbital shaking. A specification of cardiac crescents with beating protrusions was achieved at days 6–7 of culture, accompanied by the formation of yolk sac-like primitive erythroid progenitors and candidate EMP [76]. Endothelial markers were detected from day 5, with early haemogenic markers CD41 and CD45 present at day 7 (Figure 2A) with the emergence of a candidate HE [76]. In parallel, we [77] developed a longer protocol, in which the initial pulse of Activin A and CHI99021 is followed by continuous presence of VEGF and FGF2 up to day 7 and an additional 2 days of VEGF (Figure 2B). In addition to these cytokines, we tested the effect of signals implicated in haemogenic patterning in the dorsal aorta [88], and added a pulse of Sonic Hedgehog (Shh) between days 6 and 7, followed by candidate HSC and MLP-supporting cytokines SCF, TPO and FLT3L [89–91] in the last 2 days of culture. This approach achieved a gradual, wave-like, haemogenic production, with successive specification of CD41+ EMP at day 7 and candidate CD45+ MLP/MPP at days 8 and 9. Both waves included the presence of transcriptionally distinct candidate HE clusters by single-cell RNA-seq [77]. The CD45+ cells, some which co-expressing KIT and suggestive of early progenitor cells, could be visualised in small clusters budding from endothelial-lined structures [77], in a tissue organisation reminiscent of the intra-aortic clusters in which HSCs and haematopoietic progenitor cells (HPCs) are formed [12]. Importantly, signatures of niche components namely PDGFRA mesenchyme [92] and neural crest derivatives [32] could be detected transcriptionally, suggesting that day 8–9 gastruloids may contain the necessary elements to support HPC, pre-HSC and/or HSC emergence upon further maturation and tissue organisation.

## Pitfalls and potential opportunities for haemogenic gastruloid optimisation

Current haemogenic gastruloid protocols can at least partially recapitulate essential events in developmental haematopoiesis, including specification of HE and multi-wave haemopoietic cell production, with the putative generation of haematopoietic clusters. The ability to recapitulate temporal and spatial events in a single, self-organising culture system, in the absence of genetic manipulation, is an improvement on current protocols, and brings some promise of translatability of a scalable system. However, while the 216 h-protocol progresses blood cell generation to lympho-myeloid production, it stops short of HSC specification, as no long-term serial transplantation was demonstrated, and it is currently unclear whether this can be achieved.

Analysis of early developmental events in gastruloid models suggests that patterning in the presence of artificial extracellular matrices such as Matrigel may bias mesodermal output [75], as well as induce tissue (e.g. somitic) organisation that may be relevant to the structuring of vascular networks [80]. Similarly, Lutolf and collaborators generate embryonic blood and cardiac structures [76,78] in cultures subject to orbital shaking, highlighting a potential role of shear stress. Mechanical aspects are indeed central to HSC emergence (see below). *In vivo*, HSCs later move between different niches where they proliferate and/or mature, suggesting that optimisation of culture conditions and cell–cell interactions, in addition to mechanical cues, may improve the haematopoietic output of gastruloids.

**Figure 3.**
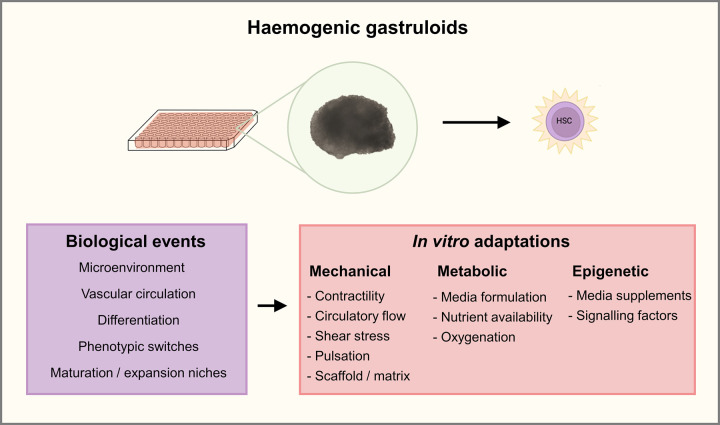
Summary of biological and experimental considerations for successful haemogenic gastruloid protocols. Production of haematopoietic stem cells (HSC) from *in vitro* haemogenic gastruloid protocols rely on the recapitulation of key biological events in haematopoiesis. Different types of *in vitro* adaptations can improve current protocols by tackling mechanical, metabolic and epigenetic characteristics of physiological processes.

In the next sections, we will consider mechanical contributions to HSC formation, as well as review metabolic and epigenetic aspects of HSC maturation in the embryo which may impact the optimisation of *in vitro* gastruloid-based protocols of HSC production (Figure 3).

### Mechanical aspects of blood development

HSCs emerge in an arterial niche shortly after the onset of circulation (E10.5 in mouse, CS10 in human), in a mechanical environment that differs from that experienced by yolk-sac waves of blood production [93,94].

Early work from the Zon laboratory proposed a requirement for cardiac contractility and the consequent circulatory flow in the generation of functional HSC from the dorsal aorta [95]. However, it remains unclear whether blood flow participates directly in HSC specification from HE, or if it impacts downstream HSC maturation and differentiation capacity [95]. Data from the Mikkola laboratory suggest that the requirement of circulatory flow for HSC production may not be absolute [96]: HSC were detected in the placenta in the absence of circulation, while the frequency of CD41+ HSC precursors in the AGM dropped from 13.6 to 2.2 per embryo in the Ncx1−/− mutant, which lacks heartbeat [96], suggesting an indirect or contributory role.

Blood flow has been shown to modulate several pathways relevant to HSC development. Shear stress sensing increases extracellular adenosine levels in the endothelium via the cAMP–PKA–CREB pathway, which causes up-regulation of BMP- and CXCL8-related genes [97,98], and can be activated by prostaglandin E2 (PGE2) [99,100]. PGE2 has been shown to expand HSC numbers *in vivo* in zebrafish, as well as from human cord blood *in vitro* [99,101]. Inducing shear stress in AGM-derived cells *ex vivo* has been shown to increase haematopoietic gene expression, together with prostaglandin, Wnt and Notch genes [100]. Additionally, the Rho-Yap mechanism has been indicated to be induced by cyclic stretch via mechano-transduction, which results in the promotion of HSPC production [102].

Engineering of pulsatile flow and shear stress in gastruloid models or other *in vitro* systems may promote the maturation of pre-HSC precursors into functional HSCs. Organisation of an arterial vascular network may further enhance the effects of flow to mimic a circulatory system. Building from the organisation of somitogenesis [80], it may be possible to embed the gastruloid in a supporting matrix to promote the extended vascular organisation. The presence of a vascular network may in turn provide a scaffold for the topographical arrangement of other cell types, for example to promote contacts between AGM-like stromal cells and nascent HSPCs, as observed to steer HSC division [103–106].

### Metabolic contributions to blood development

Mechanistic analysis of the contribution of blood flow to HSC specification or maturation in the AGM has linked it to metabolic reprogramming [103]. Interference in metabolic pathways, particularly relating to oxidative reactions and respiration, has in fact been shown to adversely affect haematopoiesis [107–110]. Embryos devoid of circulation capacity are capable of producing HE and pro-HSC, but not mature HSC due to the failure to down-regulate glycolysis to induce oxidative phosphorylation [111]. Indeed, the onset of circulation is associated with major changes in oxidative reactions, which temporally coincide with the definitive specification of HSCs; but whether circulation precedes or consolidates HSC maturation remains a topic of debate [95,112–114].

Pre-circulation embryos reside in hypoxic conditions, with glycolysis the preferred energy source for early haematopoiesis [115,116]. During EHT, cells undergo a metabolic shift from glycolysis to oxidative phosphorylation. Experimental evidence shows that modulation of pyruvate metabolism by promoting either glycolysis or oxidative phosphorylation can direct differentiation towards primitive or definitive haematopoiesis, respectively [117]. Specifically, in a human iPSC-based haematopoietic differentiation protocol, depletion of pyruvate from mitochondria to favour glycolysis skews cells towards primitive erythropoiesis, via LSD1 epigenetic control. Conversely, the influx of pyruvate promoted mevalonate and cholesterol metabolism that resulted in definitive haematopoiesis through activation of Notch1 [117]. This is in agreement with the role of Notch1 in regulating cholesterol biosynthesis, which is essential for HSC maturation [118]. Equally, a differential requirement of glutamine derivatives (dimethyl-ketoglutarate and nucleosides) was also shown to influence haematopoietic fates during EHT. In glutamine-free medium, supplementation with dimethyl-ketoglutarate increased the definitive haematopoietic output (CD45+ and lymphoid cells), while both dimethyl-ketoglutarate and nucleosides were required for primitive erythroid differentiation [119].

A second critical metabolic switch occurs at the transition from foetal to adult HSC. Foetal HSCs are generally agreed to be more proliferative, and are as such more metabolically active than the predominantly quiescent adult HSCs. This high level of activity is supported by an increased level of mitochondria, higher mitochondrial functionality, and high oxygen expenditure in foetal HSCs compared with the adult counterpart [120]. Foetal HSCs also possess a better ability to respond to reactive oxygen species (ROS) and endoplasmic reticulum (ER) stresses, notably by unfolded protein response (UPR), to cope with the oxygen demand and increased protein synthesis [121–124].

Understanding metabolic changes in blood development can inform the design of haemogenic gastruloid protocols. Supplementation of culture media with metabolites such as proline and vitamins has already been shown to skew PSC differentiation [125,126], and hypoxic conditions are used in embryonic culture protocols [127,128]. Hypoxia promotes the axial patterning of gastruloids in the absence of extrinsic Wnt signalling activators, and increases cell type diversity when in conjunction with Wnt activation [129]. Different metabolic conditions, namely glucose availability or retinoic acid supplementation, have been shown to affect the axial patterning of gastruloids [130,131]. The same may apply to blood development, as glucose modifications regulate EHT to promote haematopoietic fate [132]. Nevertheless, the variability in quantitative, spatial and temporal effects of hypoxia and metabolites remains to be determined, putatively warranting extensive protocol adaptations to achieve improved haemogenic outputs from gastruloids. The nutrient availability within the embryonic environments seems to dictate the choice of metabolic pathways, which in turn direct differentiation outcomes. While differentiation can be achieved *in vitro* via exogenous compounds, recapitulation of physiological states may be key to truly capture a wider variety of cellular states and outputs, which might be needed to generate all cell types required for correct HSC specification.

### Epigenetic and transcriptional control of blood development

Similar to metabolic activity, epigenetic regulation of gene expression has also been explored in the transition switches accompanying developmental haematopoiesis and foetal to adult HSC transitions. Several epigenetic regulators have been shown to differentially affect the successive haematopoietic waves, as exemplified by EZH2 [133], LSD1 [134] and HDAC1/2 [135], whose developmental knockouts hinder EMP formation in the yolk sac, but not intra-aortic specification of progenitors and HSC.

Early analysis of foetal-to-adult transitions showed up-regulation of elements of the polycomb repressive complexes (PRC) 1 and 2 [22], which are specifically required post-natally in HSC, and control self-renewal and differentiation programmes [136–139]. ASH1L, which unlike PRC, has transcriptional activation roles, is also required for adult HSC maintenance and quiescence, indicating a fine balance of repressive and transcriptional activation activities [140]. Indeed, global analysis of the transition from foetal to adult HSC is suggestive of a gradual activation of enhancers of adult HSC genes which does not necessitate repression of foetal programmes, at least not in the same time-frame [141], but modifies gene dependencies. Individual transcription factors (TFs) are specifically required in adult or foetal HSC: SOX17 [142] and CEBPA [143] maintain foetal and adult HSC phenotypes, respectively, putatively through a cross-regulatory circuit which involves N-MYC [143]. Other TFs such as GFI1 and ETV6, as well as FOS, are specifically required for quiescence and survival of adult HSC [144]. However, both GFI1 and ETV6 participate in HE specification [145] and EHT in HSC clusters [134,146], pointing to the importance of fine epigenetic control to diversify the effects of recurrent factor usage. As an example, *Runx1* undergoes high differential chromatin looping and enhancer utilisation from pre-HE to HSC fate commitment [147], as detected by high-resolution chromatin capture, indicating the importance of locus-specific inspection of state-specific epigenetic programmes to understand and potentially affect the mechanisms of transition.

Transferring this fine level of regulation to *in vitro* models of blood development poses a major challenge. Particularly so in light of the often incomplete reset of epigenetic marks during iPS cell reprogramming [148] and the failure of current haemogenic protocols to recapitulate *in vivo* epigenetic profiles, even in the presence of supporting stroma [149]. Selection of pluripotent cells, for example of HSPC origin, on their epigenetic profiles at critical loci may assist in this task. On the other hand, correct epigenetic programming may be facilitated in gastruloid systems in which a more complete cellular, chemical and mechanical niche is present in a single structure to facilitate integration of internal and external cues.

As previously discussed, success in *in vitro* production of HSC from pluripotent sources has been limited in its efficiency and reproducibility. The observation that the foetal-to-adult HSC transition is primarily dependent on the activation of adult gene expression programmes [141] is compatible with the reported success of HSC-fate programming using enforced ectopic expression of TFs [66]. On the other hand, the low efficiency of the protocols may indicate that observations of coexistence of multiple independent routes to HSC maturation may have physiological relevance, and that these may be incompletely represented or even suppressed by current TF-led protocols. Partial, sequential transcriptional events may allow the integration of alternative external cues to consolidate cellular states and epigenetic programmes. The existence of different classes of adult HSC, with different self-renewal and differentiation potential [29,150,151], as well as the possibility of coexistence of HSC with foetal and adult HSC in FL and neo-natal BM [152], suggests that specification and/or maturation of HSC via different coexisting routes may facilitate haematopoietic function under stress in reconstitution, as well as in homeostasis.

Alternative specification and/or maturation routes, raise the possibility that stochasticity may guide HSC formation. There is evidence of stochastic gene expression for individual regulatory molecules (e.g. *Gata2*) in the HSC-producing aorta [153], and single-cell analysis of gene expression does not clearly separate candidate pure HSC clusters from progenitors [154]. On the other hand, genes in the Notch signalling pathway, which are involved in the regulation of *Gata2* expression and HSC emergence [155], have been shown to regulate cell fate and tissue patterning [156–158] through an oscillatory expression pattern that limits and canalises noise in gene expression [159]. It is possible that the underlying stochastic expression of one or more regulatory genes configures alternative molecular entry points into HSC specification, as observed for haematopoietic lineage commitment, and more generically at cell fate transitions [160,161]. Some of these entry points may allow the cells to gain or preserve HSC function, autonomously or through interaction with environmental signals, enabling a low but stable frequency of HSCs.

Given the role of signalling pathways in buffering transcriptional stochasticity to achieve stable developmental outputs [162,163], it is possible that current protocols of haemogenic specification, which employ high and constant doses of cytokines to promote defined lineage outputs, antagonise HSC emergence or maturation. Optimisation of gastruloid models to achieve correct niche cellularity and topography, and to maximise endogenous cytokine production may overcome putative restrictive effects of sustained or uncontrolled external factor addition, to facilitate homeostatic haematopoietic development and HSC specification *in vitro*.

## Concluding remarks

We have highlighted the promise and limitations of current *in vitro* approaches to produce HSC from pluripotent sources, and reviewed mechanical, metabolic and epigenetic aspects that can be applied to protocol optimisation. Additional challenges reside in the putative low frequency and transient nature of HSC specification in the embryo, which is a late event, evolutionarily adapted for delayed contribution to blood production rather than the immediate haematopoietic needs of the developing organism. Contemporaneous production of progenitors in the AGM fulfils the immediate blood cell requirements [164], and protocols may need to incorporate conditions for the selection or maturation of HSC away from a developmental progenitor ‘default’.

The ‘organotypic’ nature of gastruloids may facilitate the correct cell transitions by providing sequential environmental cues required for HSC emergence and maturation, when combined with mechanical and putatively minimised external chemical factors. In particular, the possibility to steer gastruloid cultures into generating various types of mesoderm [75,86] may allow accurate recapitulation of the spatial–temporal tissue interactions needed for the specification of the correct HSC-generating niche. At a more fundamental level, gastruloids also provide a model by which the nature and dynamics of niche interactions and cell emergence can be studied in a way more accessible than the use of embryos. In other words, gastruloids constitute a credible system to both dissect multifactorial contributions to HSC specification and to enable it *in vitro*.

Although the number and nature of HSC specified during development remain controversial [18], the low numbers of directly transplantable HSC produced per embryo in the AGM [12] highlights the need to consider the scalability of gastruloid cultures. This can involve the engineering of large-scale arrays of gastruloids for probabilistic HSC production, and/or the implementation of 3D culture conditions that support the structured development of larger aggregates initiated by a larger number of cells. Subsequent engineering of expansion and maturation niches is likely to also be required for efficient and potentially larger output HSC production. Recent successful protocols for expansion of mouse and human HSC with preservation of functional characteristics [165,166] may be successfully incorporated at later stages to achieve HSC production at a scale that is transferrable to the clinic.

While most of the currently available gastruloid protocols are based on mouse ESCs, recent advances have resulted in human ESC-based gastruloids [87] and in human iPSC-based ‘somitoids’, which generate epithelialised human somites *in vitro* [81,167]. Currently, all available haematopoietic gastruloid protocols are mouse [76–78], and hence one important next step will be to transfer haematopoietic gastruloid protocols to the human. Reassuringly, current ‘regular’ human gastruloid protocols already express several haematopoietic and endothelial markers [87], suggesting that the development of human haemogenic protocols might be feasible in the near future. However, modifications of protocols to steer the specification of the correct types of mesoderm, as well as optimisations of the late stages of the protocol for HSC production and maturation will be needed before *in vitro* transplantable HSC production can be achieved with both mouse and human gastruloid protocols.

In conclusion, gastruloids are promising tools to study the tissue interactions and niche characteristics required for HSC formation *in vitro* with spatial and temporal accuracy. Since they are more tractable than embryos and spatially more organised than other *in vitro* platforms, gastruloid models of haematopoiesis may both improve our understanding of HSC biology, as well as tackle the challenge of *in vitro* production of transplantable HSCs in a transgene-free manner.

## Summary

- HSC production during development is a transient event dependent on chemical, mechanical and microenvironmental cues, which may require complex *in vitro* recapitulation.
- *In vitro* production of transplantable HSCs is a critical aim of regenerative medicine, which has been hindered by the lack of efficient experimental protocols.
- Gastruloid models of haematopoiesis recapitulate successive stages of developmental haematopoiesis with spatial and temporal precision, in the absence of genetic manipulation.
- Optimisation of niche reconstruction and introduction of mechanical cues provide opportunities for improved haematopoietic production in gastruloids, which may lead to the production of HSCs *in vitro*.

## Abbreviations

AGM: aorta–gonad–mesonephros
BM: bone marrow
EHT: endothelial-to-haematopoietic transition
EMP: erythro-myeloid progenitors
ESCs: Embryonic stem cells
FL: foetal liver
HE: haemogenic endothelium
HPCs: haematopoietic progenitor cells
HSC: Haematopoietic stem cells
HSPCs: hematopoietic stem and progenitor cells
IM: intermediate mesoderm
iPSC: induced pluripotent stem cells
LPM: lateral plate mesoderm
MLPs: myelo-lymphoid progenitors
MPPs: multipotent progenitors
PDMS: polydimethylsiloxane
PRC: polycomb repressive complexes
TFs: transcription factors
